# Contrast-Enhanced Ultrasound (CEUS) as an Ancillary Imaging Test for Confirmation of Brain Death in an Infant: A Case Report

**DOI:** 10.3390/children9101525

**Published:** 2022-10-05

**Authors:** Peter Slak, Luka Pušnik, Domen Plut

**Affiliations:** 1 Clinical Radiology Institute, University Medical Centre Ljubljana, Ljubljana 1000, Slovenia; 2 Faculty of Medicine, University of Ljubljana, Ljubljana 1000, Slovenia

**Keywords:** contrast-enhanced ultrasound, head ultrasound, brain death, infants, ancillary test

## Abstract

The practices for determining brain death are based on clinical criteria and vary immensely across countries. Cerebral angiography and perfusion scintigraphy are the most commonly used ancillary imaging tests for brain death confirmation in children; however, they both share similar shortcomings. Hence, contrast-enhanced ultrasound (CEUS) as a relatively inexpensive, easily accessible, and easy-to-perform technique has been proposed as an ancillary imaging test for brain death confirmation. CEUS has established itself as a favourable and widely used diagnostic imaging method in many different areas, but its application in delineating brain pathologies still necessities further validation. Herein, we present a case report of a 1-year-old polytraumatised patient in whom CEUS was applied as an ancillary imaging test for confirmation of brain death. As CEUS has not been validated as an ancillary test for brain death confirmation, the diagnosis was additionally confirmed with cerebral perfusion scintigraphy.

## 1. Introduction

Brain death is characterised by the complete and irreversible loss of brain functions, defined by the cessation of cortical and brainstem activities. The clinical criteria for the diagnosis in children were set in 1987, then updated in 2011 [1]. For the confirmation of brain death, the clinician must identify the underlying causes and determine their irreversibility. Conditions such as intoxication, hypotension, hypothermia, and metabolic/electrolyte disorders that could affect the neurologic examination have to be corrected before making the diagnosis. There is no global consensus regarding confirmatory tests [2]. The confirmation is typically made by verifying three criteria: unconsciousness, the absence of brainstem reflexes, and the apnoea test. As the latest is normally more difficult to implement in infants and can potentially cause harm in circulatory unstable patients, there is a variety of ancillary imaging tests that can assist in making the diagnosis [3].

The gold standard as an ancillary imaging test is cerebral angiography, as it confirms the cessation of cerebral circulatory blood flow. Nonetheless, this technique has several shortcomings: it is cumbersome, not always readily available, and potentially harmful, as it can exacerbate patients’ hemodynamics. Cerebral angiography, along with radionuclide scanning, remain the most used ancillary imaging tests in infants. They both, however, require trained professionals to interpret the results and can be challenging to perform in a patient who needs to be transported from an intensive care unit [4]. An additional imaging test to confirm cerebral circulatory arrest is Doppler ultrasonography (US). With Doppler US, the blood flow through intracranial and extracranial arteries can be evaluated. This method can be performed at the bedside, is cost-efficient, and poses less risk than angiography. The major setback of this technique can be transmission problems, as inadequate penetration of US beams through the temporal bone in some subjects does not always allow a reliable evaluation of intracranial vessels [5,6].

Contrast-enhanced ultrasound (CEUS) is a relatively new technique with emerging application potential that is performed with a US scanner and requires the administration of US contrast agents. US contrast agents are extremely safe, with no cardio-, hepato-, or nephrotoxic effects and with a low incidence of side effects [7]. The most widely utilised contrast agents are made up of inert gas microbubbles that are stabilised by protein or lipid shells, with a size smaller than the red blood cells. In response to the exposition to the US beam, the microbubbles resonate—that is, enlarge and shrink—and, with that, send nonlinear signals back to the transducer. Intravenously administered US contrast agents remain confined within the blood pool without diffusion into the interstitial space, including in the setting of intracranial imaging. The US scanner detects circulating microbubbles as strong echoes moving within the vessels in real-time, providing micro- and macrovascular information used to assess vascular perfusion of the whole brain [8,9]. Therefore, CEUS allows better visualisation of the cerebral vasculature in comparison to Doppler US and the evaluation of cerebral perfusion [7,10,11]. Studies in adult populations have shown that the rate of nonconclusive Doppler US examinations for determining cerebral circulatory arrest significantly reduces if CEUS is performed [6,12]. Herein, we present a case of a 1-year-old polytraumatised patient in whom CEUS was used as an ancillary imaging test for confirmation of brain death. 

## 2. Case Report

**Figure 1:**
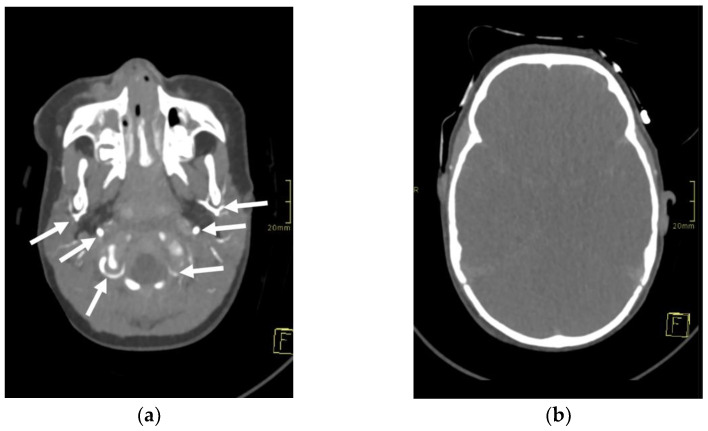
Axial 3-mm-slab maximum intensity projection of the brain computed tomography angiography (CTA) of the 1-year-old infant. The CTA images (**a**,**b**) demonstrate absent contrast opacification of the intracranial arteries. Note the normal opacification of the extracranial arteries (arrows).

A 1-year-old girl, involved in a traffic collision, was found asystolic with dilatated and nonreactive pupils. The paramedics immediately initiated cardiopulmonary resuscitation, and the patient was transferred to the nearest hospital. At arrival, the girl was still unconscious and asystolic. After 20 min, cardiac action was re-established. On physical examination, numerous wounds and contusions were found on the patient’s head, and bleeds from the nose and ears were observed. Additional contusions were noted on the trunk and extremities. The girl did not react to painful stimuli and had a Glasgow coma scale assessment score of 3. A complete blood count showed low haemoglobin (80 g/L) with elevated serum lactate levels (14 mmol/L). Whole-body computed tomography (CT) was performed forthwith. A head CT disclosed several fractures of the frontal and occipital bones, bilateral subarachnoid haemorrhage in the frontal region, and massive haemorrhage in the right maxillary sinus. No changes were noted in the cervical spine. CT of the chest and abdomen showed multiple contusions of the lungs and haematomas in the mediastinum and right inguinal region. For the treatment of severe hypotension (45/35 mmHg), the patient received blood transfusions, norepinephrine, and dopamine. The patient was transferred to a tertiary hospital centre. As intracranial hypertension (ICP = 40 mmHg) was persisting, treatment with mannitol and analgosedation was initiated. Therapeutic hypothermia was not induced due to the presence of coagulopathy. A follow-up CT scan of the head was performed approximately 12 h after the accident and showed marked cerebral oedema with completely displaced ventricles. The subarachnoid haemorrhage was more extensive, and pronounced transtentorial herniation through the foramen magnum was noted. CT brain angiography disclosed absent blood flow within the cerebral arteries (Figure 1). The patient’s condition continuously deteriorated despite intensive treatment. Due to the poor prognosis, it was decided that further treatment was not feasible, and formal tests to confirm brain death were conducted. 

**Figure 2:**
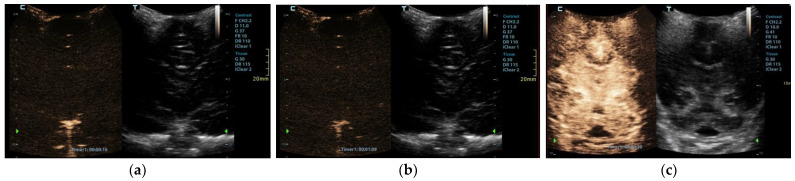
Midcoronal contrast-enhanced ultrasound images of a 1-year-old infant’s brain obtained (**a**) 10 s and (**b**) 69 s after the contrast administration. Both images demonstrate a lack of enhancement of the intracranial vasculature and no brain perfusion. (**c**) Normal CEUS brain scan in another infant is shown as a comparison.

**Figure 3:**
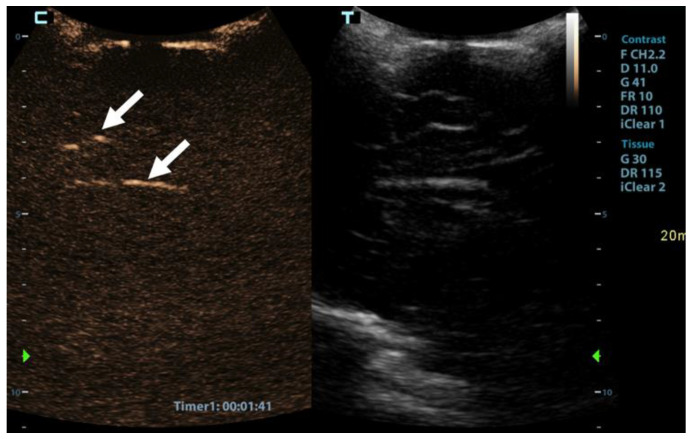
Sagittal contrast-enhanced ultrasound image of a 1-year-old infant’s brain obtained 101 s after contrast administration. The image demonstrates a scant amount of contrast microbubbles within the pericallosal artery and its branch (arrows) and no brain perfusion.

CEUS was performed as an ancillary imaging test for the confirmation of brain death. Before the examination, the details of the examination and the risks for the patient were explained to the child’s guardian. Written informed consent was obtained from the child’s guardian to perform the brain CEUS scan. The examination was performed by a paediatric radiologist with 2 years of subspecialty experience in paediatric brain imaging and 4 years of experience in performing CEUS examinations. For the examination, a Mindray M9 ultrasound scanner with a 1.4–5.1 MHz convex ultrasound transducer was used (Mindray, Shenzhen, China). SonoVue (Bracco, Milan, Italy) was used as the contrast agent. The anterior fontanelle was used as the acoustic window to scan the brain in the coronal and sagittal planes. Firstly, pre-contrast grey-scale imaging was performed to optimise the image. After that, a contrast-specific imaging mode and a low dynamic mechanical index (MI) of 0.06–0.07 was used for the scanning during the CEUS examination. To enable the simultaneous attachment of the US contrast agent and saline to the line, to avoid any delays in flushing the line with a saline flush, a three-way stopcock was connected to the existing peripheral intravenous line. At the start of the examination, 0.3 mL of US contrast agent, followed by a saline flush, was intravenously applied through a peripheral line. Only one bolus of US contrast agent was administered during the examination. For the first 60 s after the contrast administration, a continuous cine clip was obtained in the coronal plane at the level of the third ventricle, including the frontal horns of the lateral ventricles and heads of the caudate nuclei bilaterally. After that, intermittent images were obtained during the next 10 min in order to assess brain perfusion and avoid excessive contrast microbubble destruction from continuous imaging. The CEUS examination showed enhancement of the extracranial vessels and a lack of enhancement of the intracranial vessels (Figure 2). Only a scant amount of contrast microbubbles was observed within the left middle cerebral artery and pericallosal artery during the examination (Figure 3). After 10 min, we observed complete microbubble clearance and finished with the examination. The brain CEUS examination was performed in a paediatric intensive care unit at the bedside, and the whole procedure, including preparation, lasted approximately 15 min. No adverse effects were observed after the intravenous application of US contrast agent.

**Figure 4:**
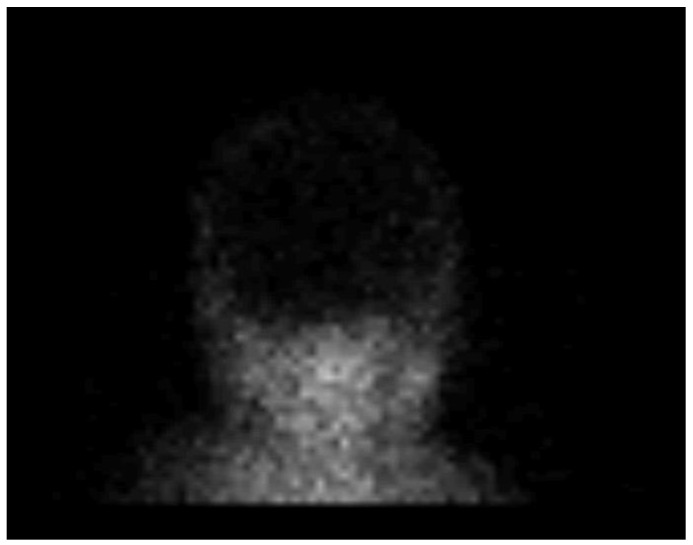
Radionuclide brain scan in the 1-year-old infant. Brain scintigraphy shows the absence of cerebral perfusion in supratentorial and infratentorial areas consistent with the diagnosis of brain death.

After the US examination, cerebral perfusion scintigraphy was performed as a widely accepted ancillary imaging test for brain death confirmation. The perfusion scintigraphy showed no accumulation of the radionuclides in the brain or the brainstem (Figure 4). 

## 3. Discussion

We presented a case of a polytraumatised infant with foraminal herniation of the cerebellar tonsils in whom CEUS was employed as an ancillary imaging test for the confirmation of brain death. As CEUS is not a validated ancillary test for brain death conformation, the diagnosis was additionally confirmed with cerebral perfusion scintigraphy.

In recent years, CEUS has established itself as a beneficial and widely used diagnostic imaging method in many different areas, such as echocardiography, for the evaluation of vesicoureteral reflux and characterisation of liver neoplasms [10]. Due to a lack of clinical reports and safety studies, the use of CEUS for the assessment of brain pathology in children is, for now, still considered off-label [11,13]. Nevertheless, there has been an increasing number of reports on the use of CEUS in children for the assessment of hypoxic-ischemic injury, acute ischemic stroke, brain tumours, paediatric neurovascular diseases, epilepsy, and the confirmation of brain death [5,11,14,15]. These reports validate CEUS as a great method for the evaluation of the brain in children, especially the brain vasculature and vascular pathologic processes. We found only one report where CEUS was used as an ancillary imaging test for the confirmation of brain death in an infant. In this report by Hwang et al., the neonate passed away before they could confirm brain death with a formal radiologic evaluation. Therefore, in their case, the CEUS findings could not be compared to a validated imaging method [5]. In the case we presented, the absence of brain perfusion was confirmed by perfusion scintigraphy. 

CEUS allows the observation of organ perfusion over a longer period of time, which makes it a great tool for the evaluation of organ perfusion. It takes up to 15 min for the contrast microbubbles to be metabolised and disappear from the vessels [16,17]. In our case, we observed the brain for 10 min. During this time, we did not observe any constant flow through any of the intracranial arteries. However, during the observation period, a scant of contrast microbubbles were observed within the left middle cerebral artery and pericallosal artery for a few seconds. It is important to note that, although we observed scant contrast microbubbles within the intracranial arteries, the perfusion scintigraphy showed no accumulation of the radionuclides in the brain. Therefore, we can assume that scant microbubbles within the intracranial arteries can be a finding on CEUS during the test for brain death confirmation and that this finding does not exclude a brain death diagnosis. However, as this was the first such examination that we performed and no other similar reports exist in the literature, we cannot make any final conclusions. In the aforementioned case by Hwang et al., they reported “near absent perfusion of the intracranial vessels”, which is similar to our case [5]. Further research will be needed to better delineate the extent and context of scant microbubbles within intracranial arteries for this kind of test.

The safety profile of US contrast agents has been documented in numerous studies in adult and paediatric populations. The US contrast is considered safe, with a comparable or lower risk of adverse events to the MRI contrast agents and almost 70-times lower risk compared to the iodinated CT contrast agents. Furthermore, it can be used in the case of impaired renal function, as it is not nephrotoxic [7]. An additional advantage of this technique is also that the administration of the US contrast agent can be repeated within the same setting if, for any reason, an examination fails or the results are not entirely clear after the first bolus. There is still limited data on the contrast safety in the paediatric population, with individual reports and questionnaire-based surveys reporting mild transitory adverse effects such as skin reactions, hypoventilation, altered taste, light-headedness, and transient tinnitus [18,19]. Nonetheless, in general, the US contrast is considered to have a favourable safety profile [20]. In our case, no adverse effects after intravenous application of the US contrast agent were observed.

In the adult population, the transcranial Doppler US is considered one of the main ancillary tests for the confirmation of brain death [21]. For the transcranial Doppler US, the thin temporal bone near the zygomatic arch bilaterally is usually used as the acoustic window (i.e., transtemporal window), although the suboccipital acoustic window can also be used. If a diastolic retrograde blood flow, systolic spikes, or completely absent blood flow in the main intracranial arteries are observed, this confirms the cerebral circulatory arrest [22]. Nevertheless, the transtemporal window does not always allow good visualisation of the intracranial arteries. Welschehold et al. recently demonstrated that transcranial Doppler US was not always feasible in approximately a quarter of their adult subjects [6]. Several researchers turned to CEUS with the transcranial Doppler US technique to achieve a better visualisation of intracranial arteries. These studies performed on the adult population show that the rate of nonconclusive transcranial Doppler US examinations for determining cerebral circulatory arrest significantly reduces if the US contrast agent is applied [6,12].

In our opinion, CEUS is approaching the ideal ancillary test for the confirmation of brain death in infants. The open anterior fontanelle in infants serves as an ideal acoustic window for the assessment of brain vasculature, much better than the transtemporal window in the adult population [5,8,17,23]. CEUS is also relatively inexpensive, easily accessible, safe, and available at the bedside, with the results not susceptible to sedative medications [4]. Nevertheless, further validation is still required before CEUS can be employed as an adjunct to other ancillary tests. Such a validation of the method would be valuable, as there is still a paucity of data regarding additional ancillary imaging tests for a brain death diagnosis in children, and only a scarce number of them have been validated in the paediatric population [24].

## 4. Conclusions

We presented a case of an infant in whom CEUS was employed as an ancillary imaging test for the confirmation of brain death. The case demonstrated CEUS as a reliable, rapid, non-invasive, easy-to-perform at the bedside, and feasible technique that could serve as an ancillary imaging test for brain death confirmation in infants. Further comparative studies on larger cohorts are required to confirm its potential.

## Data Availability

The data supporting the findings of this study are private due to the protection of personal data.
